# Insights Into Neuroimaging Findings of Patients With Coronavirus Disease 2019 Presenting With Neurological Manifestations

**DOI:** 10.3389/fneur.2020.593520

**Published:** 2020-11-09

**Authors:** Boran Chen, Chaoyue Chen, Junkai Zheng, Ruoyu Li, Jianguo Xu

**Affiliations:** ^1^Department of Neurosurgery, West China Hospital, Sichuan University, Chengdu, China; ^2^West China School of Medicine, Sichuan University, Chengdu, China; ^3^State Key Laboratory of Biotherapy and Cancer Center, West China Hospital, Sichuan University, Chengdu, China; ^4^Collaborative Innovation Center for Biotherapy, Chengdu, China

**Keywords:** COVID-19, SARS-CoV-2, neuroimaging, CT, MRI, ischemic infarction, cerebral hemorrhage, white matter abnormality

## Abstract

**Objective:** This mini review aims to provide insight into the neurological imaging in patients with coronavirus disease 2019 (COVID-19).

**Methods:** PubMed, Embase, and Web of Science were searched through July 21, 2020, for relevant studies reporting the neuroimaging findings in COVID-19 patients with neurological manifestations. Proportion estimates with a 95% confidence interval (CI) were pooled after the Freeman–Tukey transformation. The heterogeneity across the included studies was also assessed.

**Results:** Overall, 11 studies with a total of 659 patients were included. The pooled proportion estimate of abnormal neuroimaging finding in patients who exhibited neurological manifestation and underwent brain CT or MRI was 59% (95% CI, 39–77%). The proportions of acute/subacute ischemic infarction, intracranial hemorrhage, and subcortical or deep white matter abnormalities were 22% (95% CI, 17–28%), 24% (95% CI, 17–30%), and 27% (95% CI, 12–45%), respectively.

**Conclusion:** This mini review comprehensively detailed neuroimaging findings of patients with COVID-19 and neurological manifestations. Clinicians should be familiar with the neuroimaging patterns to catch the sight of brain abnormalities caused by severe acute respiratory syndrome coronavirus 2 (SARS-CoV-2).

## Introduction

The catastrophic pandemic, coronavirus disease of 2019 (COVID-19), is caused by the severe acute respiratory syndrome coronavirus 2 (SARS-CoV-2) (1). COVID-19 has spread rapidly through countries all over the world, and the number of patients has been rising dramatically. As of October 12, 2020, over 37 million cases have been diagnosed with COVID-19 and over 1 million people have died of this disease (2).

Common symptoms caused by virus infection of the respiratory system such as fever, cough, dyspnea, and fatigue have been demonstrated by previous reports (3, 4). Most patients presented a mild course of disease and resolved without specific treatment. However, involvement of other systems frequently happened in critically ill patients, especially the central nervous system (5). With improvement of our understanding of SARS-CoV-2, an increasing number of patients with COVID-19 who exhibited neurological manifestations have been reported. In a retrospective observational study from Wuhan, China, 36% of the 214 consecutive patients diagnosed with COVID-19 had neurological manifestations (6). Early evidence showed that muscle injury or myalgia was the most common manifestation of neurological involvement with a prevalence of 19.2%, followed by headache (10.9%), dizziness (8.7%), and nausea (10.9%) (7). Uncommon neurological manifestations included ischemic stroke, intracerebral hemorrhage, myelitis, Guillain–Barré syndrome, Bell's palsy, and rhabdomyolysis (8). Brain computed tomography (CT) and magnetic resonance imaging (MRI) are two commonly used methods and have the potential to show neurological abnormalities associated with COVID-19. Herein, we performed this mini review to provide insight into the neurological imaging in patients with COVID-19.

## Methods

This mini review was conducted and reported based on Preferred Reporting Items for Systematic Reviews and Meta-Analyses (PRISMA) statement. A comprehensive literature search was processed in PubMed, Embase, and Web of Science through July 21, 2020, with the following search strategy: ((COVID-19[title/abstract]) OR (COVID 19[title/abstract]) OR (SARS-Cov-2[title/abstract])) AND ((brain[title/abstract]) OR (CNS[title/abstract]) OR (central nervous system[title/abstract]) OR (neurologic[title/abstract])). We also identified potential eligible literature by screening the reference list of included studies. Two reviewers performed the literature search independently. Disagreement was arbitrated by a third reviewer.

The inclusion criteria were: (1) reporting neuroimaging findings (e.g., brain CT and MRI findings) of patients who were diagnosed with COVID-19 and had neurological manifestations; (2) documentation of the prevalence of neuroimaging findings. Studies were excluded if meeting any of the following criteria: (1) not accessible in English; (2) patients aged <18 years or >100 years; (3) total sample size of <10; (4) not peer-reviewed article; (5) review, letter, or editorial.

We applied the 11-item scale recommended by the Agency for Healthcare Research and Quality (AHRQ) to assess the quality of included studies, which were included in the quantitative analysis. Each item was scored 1 for “YES” or 0 for “NO” or “UNCLEAR.” The quality of each study was graded as good (8–11), moderate (4–7), or bad (0–3).

Data collected were the first author, country, sample size, mean age, number and rate of male, neuroimaging tool, number, and proportion of positive neurological findings, as well as neurological manifestations. Data were extracted by two reviewers independently. Any discrepancy between the two reviewers was judged by a third reviewer.

STATA/SE 15.1 (Stata Corporation, College Station, TX) software was used to perform analyses. Proportion estimates with a 95% confidence interval (CI) were pooled after the Freeman–Tukey transformation (double arcsine formation) to stabilize the variances (9). We used a random-effect model to account for heterogeneity. The heterogeneity among included studies was determined by *I*^2^ test. *I*^2^ above 50% was considered high heterogeneity. *P* < 0.05 was considered statistically significant. Subgroup analysis was conducted by continent and neuroimaging tool to identify further potential sources of heterogeneity. We assessed publication bias by visualization of funnel plot and performing Begg's test and Egger's test (10).

## Results

**Figure 1:**
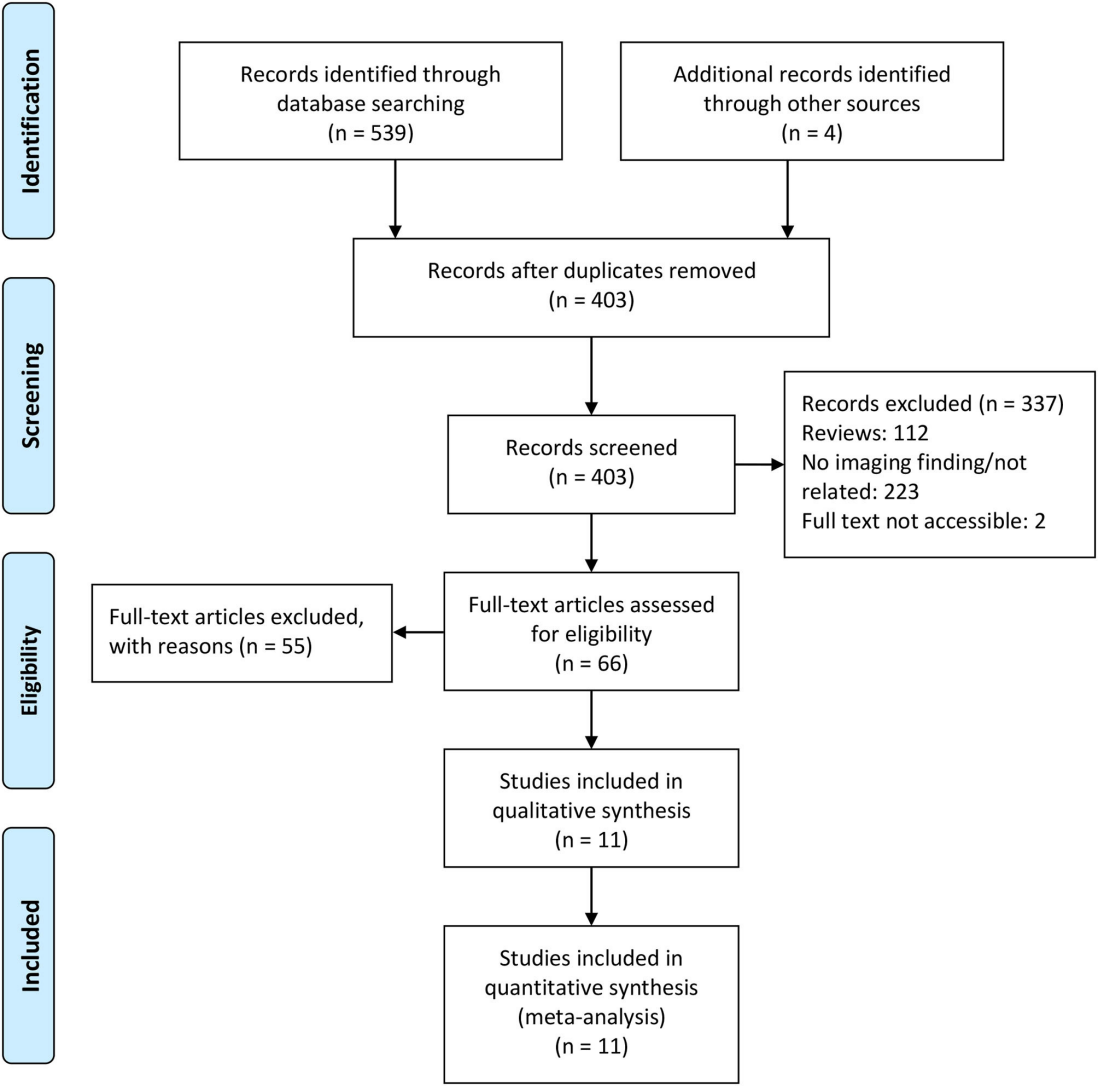
PRISMA flow diagram of literature retrieval.

The initial literature search involved a total of 403 records after adding additional records and removing duplicated records, with 11 records included in this mini review. Figure 1 shows the PRISMA flow diagram of this study.

The characteristics of the included studies are presented in Table 1. A total of 659 patients were included in the quantitative analysis. The sample sizes ranged from 19 to 242. The mean/median age of patients in each study ranged from 58.5 to 77 years old. The male rates ranged from 46 to 81%. Most of them were of medium quality. Four studies performed brain CT as the neuroimaging tool (13, 18, 20, 21). All the studies performed MRI except the study by Xiong et al. (21), which only performed brain CT in order to reduce the exposure risk of the staff.

**Table 1:** Characteristics of included studies for quantitative analysis.

**References**	**Quality (score)**	**Country**	**Sample (*n*)**	**Age**	**Male, *n* (%)**	**Neuroimaging tool**	**Positive neuroimaging finding, *n* (%)**	**Neuroimaging findings, *n* (%)**	**Neurological manifestation, *n* (%)**
Chougar et al. (11)	Medium (7)	France	73	58.5 ± 15.6	48/73 (66)	MRI	43/73 (59)	Ischemic lesion, 17 (23); CVT, 1 (1); micro-hemorrhage, 20 (28); perfusion abnormalities, 22 (48); multifocal white matter lesions, 4 (6); basal ganglion lesion, 4 (6); CLOCC, 3 (4); PRES, 2 (3); hypo-ischemic lesions, 3 (4); central pontine myelinolysis, 3 (4); meningeal enhancement, 2 (5); corticospinal tract FLAIR hyperintensity, 1 (1); neuritis, 2 (3)	Impaired consciousness, 39 (54); focal neurological deficit, 31 (43); seizure, 10 (14)
Collen et al. (12)	Medium (6)	Belgium	19	77 (range 49–94)	14/19 (74)	MRI	8/19 (42)	Hemorrhage, 2 (11); white matter changes, 4 (21); asymmetric olfactory bulbs, 4 (21)	Headache, 2 (10); agitation, confusion, disorientation, 5 (26); seizure, 1 (5)
Giorgianni et al. (13)	Bad (3)	Italy	26	70.6 (range 21–88)	12/26 (46)	CT/MRI	10/26 (38)	Ischemic lesions, 4 (15); hemorrhage, 5 (20); encephalitis, 1 (4)	Coma, 6 (23); confusional state, 4 (15); dizziness, 3 (12); headache, 1 (4); paresis, 6 (23); other, 6 (23)
Helms et al. (14)	Medium (4)	France	58	63^a^	NA	MRI	11/13 (84.6)	Cerebral ischemic stroke, 3 (23); leptomeningeal enhancement, 8 (62)	Agitation, 40 (69); corticospinal tract signs, 39 (67); dysexecutive syndrome, 14 (36)
Kandemirli et al. (15)	Medium (5)	The United States	27	63 (range 34–87)	21/27 (78)	MRI	12/27 (44)	Cortical FLAIR abnormality, 10 (37); white matter abnormality, 3 (11)	NA
Kremer et al. (16)	Medium (7)	France	64	65 (range 20–92)	43/64 (67)	MRI	36/64 (56)	Ischemic lesions, 17 (27); leptomeningeal enhancement, 11 (17); encephalitis, 8 (13)	Headache, 10 (16); seizure, 1 (2); anosmia, 2 (3); ageusia, 4 (6); corticospinal tract signs, 20 (31); impaired consciousness, 25 (39); confusion, 34 (53); agitation, 20 (31)
Kremer et al. (17)	Medium (6)	France	37	61 ± 12	30/37 (81)	MRI	37/37 (100)	Micro-hemorrhage, 9 (24); white matter abnormality, 37 (100)	Impaired consciousness, 27 (73); wakefulness after sedation, 15 (41); confusion, 12 (32); agitation, 7 (19); headache, 4 (11); seizure, 5 (14)
Pons-Escoda et al. (18)	Medium (4)	Spain	103	74 (50.2–90)^b^	63/103 (61)	CT/MRI	26/103 (25)	Ischemic lesions, 13 (13); hematoma, 8 (8); aneurysm, 3 (3); metastasis, 2 (2)	Headache, impaired consciousness, dysarthria, gait abnormality, 40 (39); stroke/TIA, 25 (24); traumatic brain injury, 17 (17); focal symptoms, 11 (11); post-sedation encephalopathy, 5 (5); seizure, 3 (3)
Radmanesh et al. (19)	Medium (5)	The United States	27	NA	NA	MRI	27/27 (100)	Ischemic lesions, 11 (41); micro-hemorrhage, 9 (33); white matter abnormality, 10 (37)	Persistently depressed mental status, 27 (100)
Radmanesh et al. (20)	Medium (6)	The United States	242	68.7 ± 16.5	150/242 (62)	CT/MRI	205/242 (85)	Chronic ischemic lesions, 47 (20); acute/subacute ischemic lesions, 13 (5); white matter abnormality, 134 (55); hemorrhage, 11 (5)	Altered mental status, 102 (42); syncope/fall, 79 (33); focal neurological deficit, 30 (12)
Xiong et al. (21)	Medium (5)	China	28	NA	NA	CT	9/28 (32)	Ischemic lesions, 6 (21); others, 3 (11)	Headache, 10 (36); stroke/history of stroke, 8 (29); occipital neuralgia, 1 (4); impaired consciousness, 1 (4); traumatic brain injury, 1 (4); syncope, 1 (4)

*RT-PCR, reverse transcriptase-polymerase chain reaction; CT, computed tomography; MRI, magnetic resonance imaging; CVT, cerebral venous thrombosis; CLOCC, cytotoxic lesion of the corpus callosum; PRES, posterior reversible encephalopathy syndrome; FLAIR, fluid attenuated inversion recovery*.

a *Median age*.

b *Fifth to 95th range*.

**Figure 2:**
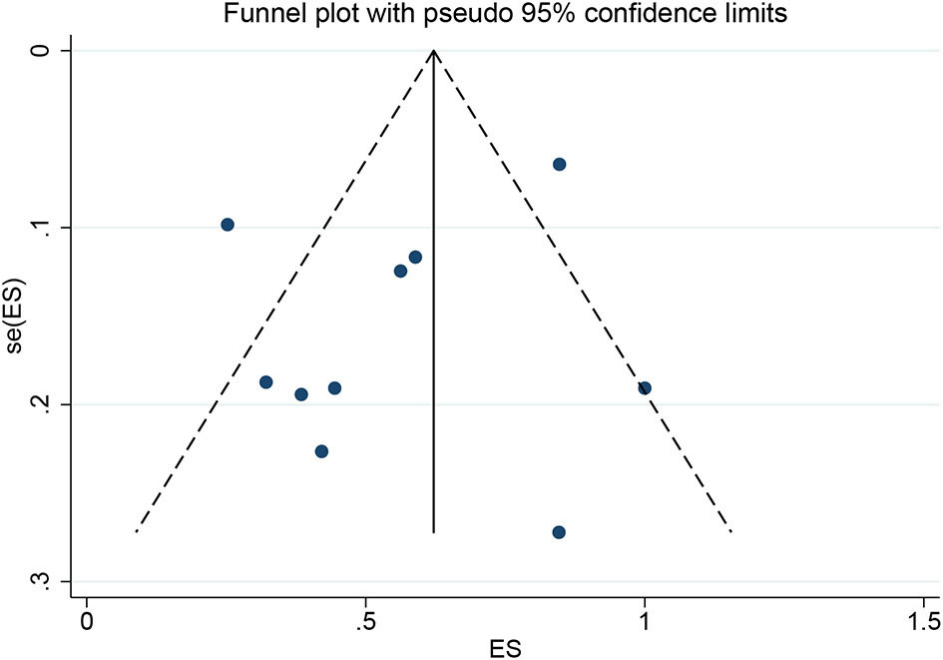
Funnel plot for the proportion of abnormal neuroimaging finding in patients who exhibited neurological manifestation and underwent brain CT or MRI.

**Figure 3:**
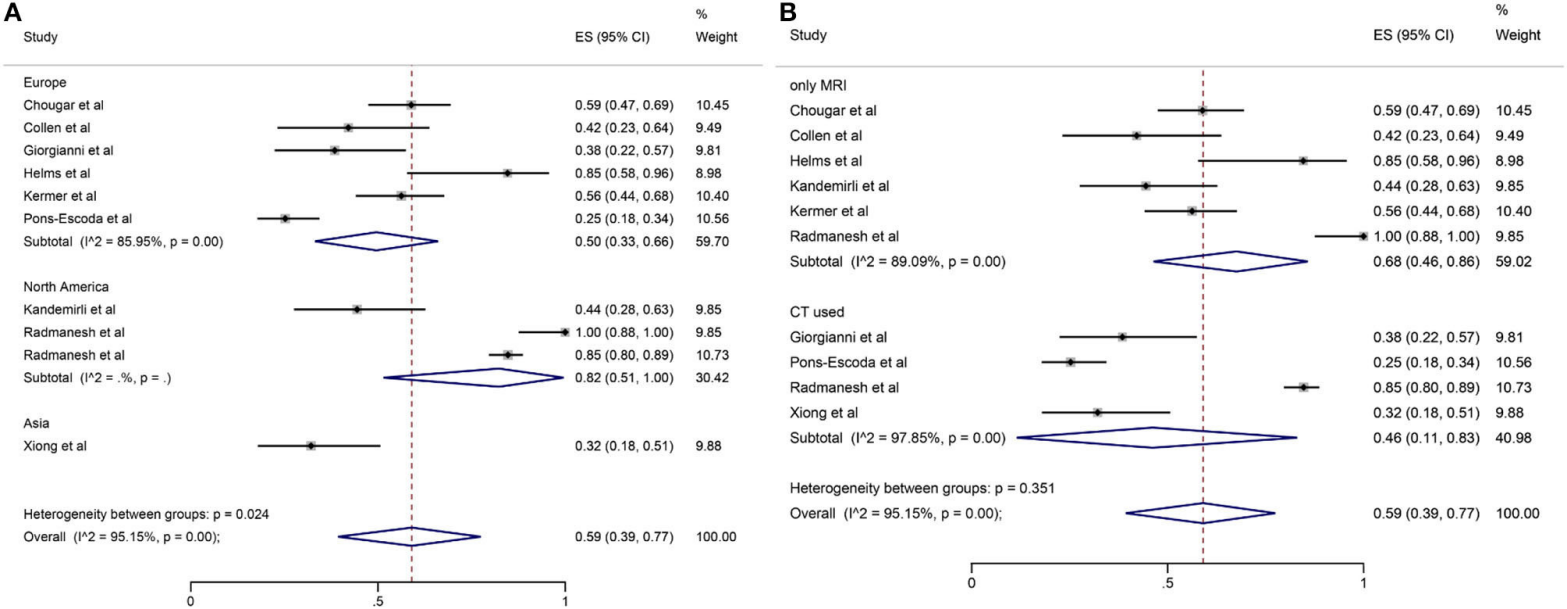
Forest plot for subgroup analysis by **(A)** continent; **(B)** neuroimaging tool regarding the proportion of abnormal neuroimaging finding.

**Figure 4:**
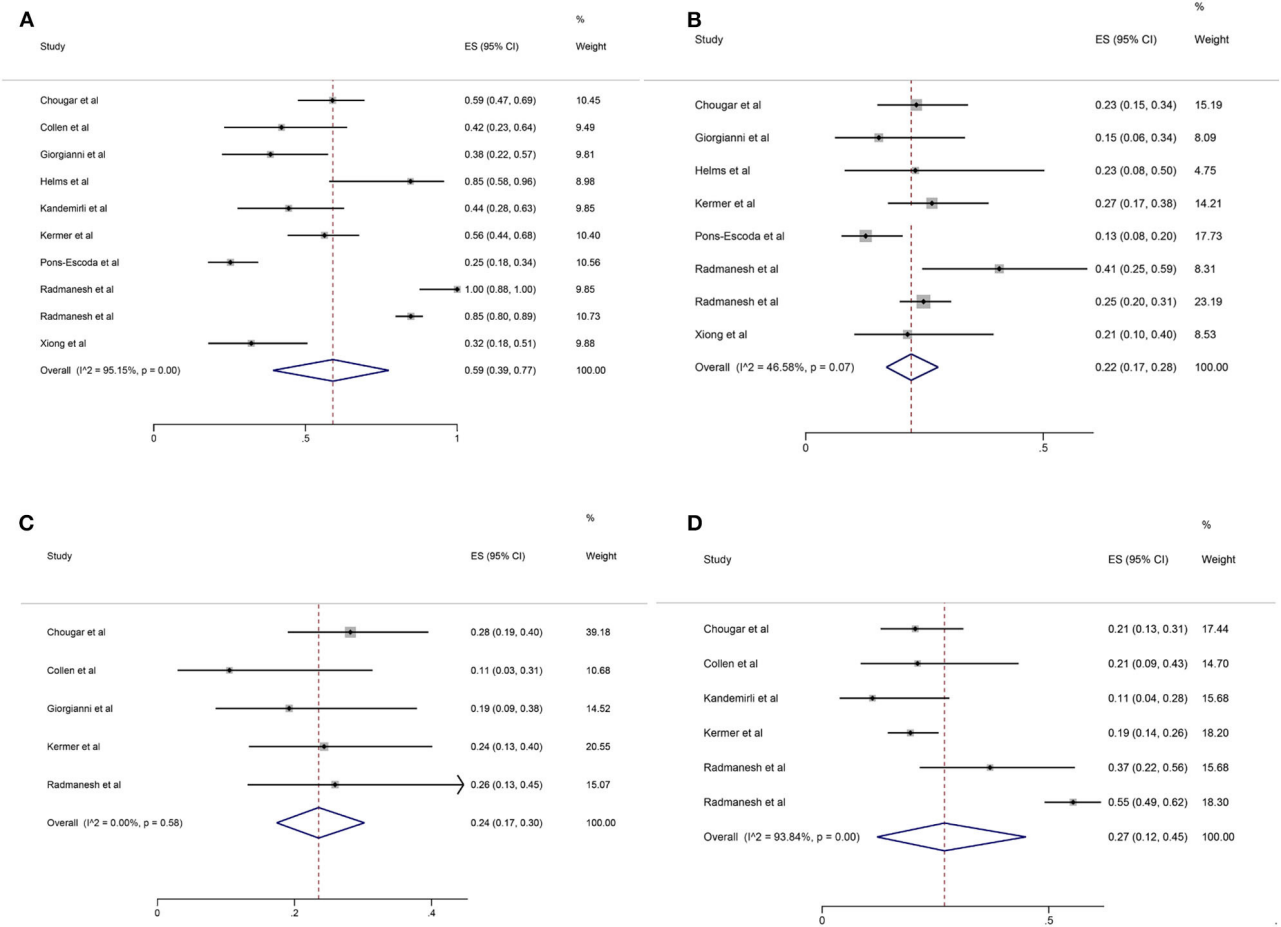
Forest plot for the proportion of **(A)** abnormal neuroimaging finding; **(B)** acute/subacute ischemic infarction; **(C)** intracranial hemorrhage; **(D)** subcortical or deep white matter abnormalities in patients who exhibited neurological manifestation and underwent brain CT or MRI.

Among the 11 included studies, 415 out of 659 patients had abnormal brain CT or MRI findings. The overall proportion estimate of abnormal neuroimaging finding in patients who exhibited neurological manifestation and underwent brain CT or MRI was 59% (95% CI, 39–77%) with a high level of heterogeneity (*I*^2^ = 95.15%) from 10 studies (**Figure 4A**). One study excluded patients with normal imaging, ischemic infarcts, cerebral venous thrombosis (CVT), or chronic lesions unrelated to the coronavirus disease. However, detailed information of excluded patients was inaccessible except data of 37 patients with white matter abnormalities. As a result, this study was excluded from the pooled analysis of abnormal neuroimaging finding proportion estimate. The funnel plot was visually symmetric, suggesting no significant publication bias (Figure 2). Additionally, analyses of Egger's test (*t* = −1.03, *p* = 0.335) and Begg's test (*z* = −0.09, *p* = 1.000) yielded evidence of no significant publication bias. We performed subgroup analysis regarding different continents (Europe, North America, or Asia) and neuroimaging tool (only MRI or used CT). The pooled proportion of abnormal neuroimaging finding was 50% (95% CI, 33–66%), 82% (95% CI, 51–100%), and 32% (95% CI, 18–51%) in Europe, North America, and Asia, respectively (Figure 3A). The pooled proportion estimates were 68% (95% CI, 46–86%) among studies that only used MRI as the neuroimaging tool and 46% (95% CI, 11–83%) among studies that used CT (Figure 3B). Acute/subacute ischemic infarctions on brain CT/MRI were reported by eight studies (11, 13, 14, 16, 18–21). The pooled proportion estimate was 22% (95% CI, 17–28%) with a low level of heterogeneity (*I*^2^ = 46.58%) (Figure 4B). Five studies reported intracranial micro- or macro-hemorrhage (11–13, 17, 19). Micro-hemorrhages, best visualized by susceptibility weighted imaging (SWI) of MRI, were defined as rounded foci <5 mm in basal ganglia or subcortical white matter that result from rupture of small vessels (19, 22). Correspondingly, macro-hemorrhages were defined as foci larger than 5 mm. The pooled result showed that the proportion of intracranial hemorrhage in patients who underwent brain CT/MRI was 24% (95% CI, 17–30%) with a low level of heterogeneity (*I*^2^ = 0) (Figure 4C). However, we were unable to perform pooled analysis on micro- and macro-hemorrhage separately due to the inconsistent reporting standards of the included studies. Six studies reported subcortical or deep white matter abnormalities (11, 12, 15, 17, 19, 20). The proportion estimate was 27% (95%CI, 12–45%) with a high level of heterogeneity (*I*^2^ = 93.84%) (Figure 4D). Results of quantitative analysis are presented in Table 2.

**Table 2:** Summary of quantitative analysis results.

**Neuroimaging finding**	**No. of studies**	**Proportion**	**95% CI**	***I*^**2**^(%)^**a**^**	***P*-value^**a**^**
Abnormal finding	10	0.59	0.39-0.77	95.15	<0.01
Acute/subacute ischemic lesion	8	0.22	0.17-0.28	46.58	0.07
Hemorrhage	5	0.24	0.17-0.30	0	0.58
White matter abnormality	6	0.27	0.12-0.45	93.84	<0.01

*CI, confidence interval*.

a *Indexes to evaluate heterogeneity among studies*.

## Discussion

In this mini review, we described neuroimaging findings on brain CT/MRI in patients diagnosed with COVID-19. Neuroimaging patterns were identified from the included studies, and their proportion was estimated. Overall, 59% of COVID-19 patients exhibited neurological manifestations and underwent brain CT or MRI had diverse neuroimaging abnormalities. Notably, about 41% of patients with COVID-19 and available neuroimaging showed normal findings. Part of the explanation was that these patients exhibited nervous system manifestations prior to detectable structural changes.

### Neurovascular Event

Ischemic lesions were located in both large and small vessels, such as middle cerebral artery (23–26), anterior cerebral artery (24), posterior inferior cerebellar artery (26, 27), pericallosal artery (28), lenticulostriate artery (26), etc. Anterior circulation artery involvement accounted for 69.2% in acute/subacute infarctions according to an American study (20). The association between large vessel stroke and COVID-19 has been underlined by recent evidence. It was hypothesized that COVID-19-related hypercoagulability could cause thrombosis and embolism, thus leading to large vessel occlusion in light of the high prevalence in the young population and the absence of vessel wall disease (29, 30). However, the mechanism of small vessel occlusion remains unclear and needs further investigation. Small cortical ischemic lesions were revealed by MRI in a COVID-19 patient while neither interstitial lung involvement nor abnormal serum inflammation markers were detected (31). Furthermore, a multicenter retrospective study comparing COVID-19 patients with non-COVID-19 patients from 1 year prior found that infection with COVID-19 was the strongest independent risk factor for stroke, followed by deep vein thrombosis and male sex (32).

Micro-hemorrhages are best visualized by SWI, presenting as hypoattenuating foci. It was reported that intensive care unit (ICU) patients were more likely to experience micro-hemorrhage compared with non-ICU patients (11). De Stefano et al. (33) reported the first case of a critically ill patient with COVID-19 with massive multifocal parenchymal micro-hemorrhage on SWI while no parenchymal lesion was observed on T1- or T2-weighted imaging. A case series described unusual micro-hemorrhage distribution, which was located in the corpus callosum, internal capsule, and middle cerebellar peduncles, in nine ICU patients (34). Macro-hemorrhage was also reported. In a COVID-19 patient with altered mental status, bilateral ganglia macro-hemorrhages were observed on both CT and MRI, which was absorbed shown by a 7-day follow-up CT (35). As is known, the amount of intracranial hemorrhage has a great effect on the outcome of patients (36). However, no research has explored the effect of number or amount of intracranial hemorrhage in the setting of COVID-19.

### White Matter Abnormality

We found that white matter abnormalities was the most frequent neuroimaging pattern in patients with COVID-19 and neurological manifestations, which is consistent with a previous review (37). White matter-specific injuries was presented in a case series of six COVID-19 patients who exhibited an altered mental status (38). MRI revealed FLAIR hyperintensities in bilateral deep white matter in all six patients, corpus callosum in one patient, middle cerebellar peduncles in five patients, and corticospinal tracts in three patients (38). A case series reported four children infected by SARS-Cov-2 with mild respiratory symptoms and neurological symptoms such as encephalopathy and proximal muscle weakness, and the common neuroimaging pattern was hypointensity on CT and hyperintensity with restricted diffusion on T2 MRI in the splenium of the corpus callosum, known as cytotoxic lesion of the corpus callosum (CLOCC), which is a rare but reversible lesion (39). Anzalone et al. (40) reported four COVID-19 cases with minimum involvement in the adjacent subcortical white matter, who presented neurological symptoms of agitation and spatial disorientation. However, MRI findings were predominated by multifocal cortical signal changes in this case series, which was presented as hyperintensities on T2 and FLAIR MRI (40). In summary, white matter abnormalities were presented as confluent hyperintensities on T2/FLAIR of MRI with abnormal restricted diffusion and hypointensities on CT and T1-weighted imaging in subcortical and deep white matter, as well as corpus callosum, middle cerebellar peduncles, and corticospinal tracts, causing non-specific neurological signs.

### Other Abnormal Neuroimaging Findings

Other neuroimaging findings included leptomeningeal enhancement, cortical abnormalities, smaller olfactory bulb, and abnormal peripheral nerves. Leptomeningeal enhancement was depicted by post-contrast T1W1 or FLAIR images and better visualized by delayed post-contrast FLAIR (15, 16). Agitation was a frequent neurological symptom for patients with leptomeningeal enhancement (16). Like white matter signal abnormalities, cortical signal abnormalities were presented as increased FLAIR and diffusion-weighted signal (15). Additionally, the distribution of cortical abnormalities was non-specific and could affect all lobes (15). Anosmia is a common neurological symptom of COVID-19 (41). The neuroimaging findings of COVID-19 patients with anosmia involved asymmetric olfactory bulbs on spin echo MRI, hyperintensities inside bilateral olfactory bulbs on T2 MRI with fat suppression and FLAIR, as well as normal MRI images (42–44). Emerging evidence indicates that SARS-CoV-2 can trigger autoimmune neurological diseases such as Guillain–Barré syndrome (45). In a COVID-19 patient with bifacial weakness and paresthesia subtype Guillain–Barré syndrome, abnormal enhancement of oculomotor nerve, abducens nerves, and facial nerves was shown by post-contrast T1 MRI (46).

### Association Between Coronavirus Disease 2019 and Abnormal Neuroimaging Findings

Evidence has shown that patients with severe COVID-19 were more likely to have abnormal neuroimaging findings. In a retrospective COVID-19 study, patients with leukoencephalopathy or cerebral micro-hemorrhages had higher peak D-dimer levels and peak international normalized ratio, lower Glasgow Coma Scale and nadir platelet count, longer ventilation time, and hospitalization, more severe acute respiratory distress syndrome, and worse functional status on discharge compared with patients who had normal brain MRI findings (47). When comparing COVID-19 patients with non-COVID-19 patients, increased frequency of in-hospital stroke onset was found in the former cohort (48 vs. 5%) (32). Results of another study revealed that patients with COVID-19-related ischemic stroke had worse functional outcome and higher mortality than patients with ischemic stroke and without COVID-19 (48). However, no study has compared the frequency or outcome of neuroimaging findings other than stroke between COVID-19 and non-COVID-19 patients.

### Limitations

This study has several limitations. First, in order to accelerate the process, our initial search was restricted to [Title/Abstract] in consideration of the urgency of this pandemic. It is possible that we have left out some related literature. Nevertheless, we screened the reference list of included studies to identify potential eligible studies. Second, our quantitative analysis included patients who only underwent brain CT. As a result, the pooled proportion of neuroimaging findings might be underestimated. Third, the studies included in this mini review were retrospective observational studies with an inherent restriction on reliability. Besides, most of the studies included in the quantitative analysis were of medium quality. However, due to the pandemic of COVID-19, high-quality researches are difficult to conduct. Fourth, we were unable to quantify the extent of white matter changes. Further studies reporting quantitative indices such as Fazekas scale are required. Fifth, neuroimaging findings may not be related to COVID-19. Aging is known to have a relationship with structural and functional brain changes. The phenomenon of frequent hyperintensities on MRI in older adults is well-established, especially in white matter (49). This change is usually caused by the loss of myelin sheets and axonal fibers resulting in impaired white matter integrity (50). As mentioned above, mean age of patients in the studies ranged from 58.5 to 77 years old. As a result, we can reckon that a large percentage of the patients included in our analysis were older patients. There is an inevitable question of whether the neuroimaging changes, especially white matter abnormalities, were COVID-19-related or age-related.

## Conclusion

This mini review comprehensively detailed neuroimaging findings of patients with COVID-19 and neurological manifestations. Although the mechanism of neurological involvement in patients with COVID-19 has not been clearly interpreted, clinicians should be familiar with the neuroimaging patterns to catch the sight of brain abnormalities caused by SARS-CoV-2. Further research investigating the pathophysiology of neurological abnormalities, the association between abnormal neuroimages and clinical outcomes, as well as long-term follow-up of these patients is warranted to better understand the process of neuropathology and to manage patients with neurological changes in the setting of COVID-19.

## Author Contributions

BC and CC: study concept and design, literature review, data extraction, writing of the initial draft, and final revision. JZ: analysis, interpretation, and writing of the initial draft. RL: analysis and interpretation. JX: supervision. All authors: contributed to the article and approved the submitted version.

## Conflict of Interest

The authors declare that the research was conducted in the absence of any commercial or financial relationships that could be construed as a potential conflict of interest.
